# Intravitreal Injection of ZYAN1 Restored Autophagy and Alleviated Oxidative Stress in Degenerating Retina via the HIF-1α/BNIP3 Pathway

**DOI:** 10.3390/antiox12111914

**Published:** 2023-10-26

**Authors:** Xiao-Na Hao, Na Zhao, Jie-Min Huang, Si-Yu Li, Dong Wei, Ning Pu, Guang-Hua Peng, Ye Tao

**Affiliations:** Department of Physiology and Neurobiology, Laboratory of Visual Cell Differentiation and Regulation, School of Basic Medical Sciences, Zhengzhou University, 100 Science Avenue, Zhengzhou 450001, China; haoxn0126@163.com (X.-N.H.); zn0114@gs.zzu.edu.cn (N.Z.); huangjm1217@163.com (J.-M.H.); lsiyu@gs.zzu.edu.cn (S.-Y.L.); 202047000423@stu.zzu.edu.cn (D.W.); 202051081418@stu.zzu.edu.cn (N.P.)

**Keywords:** retina, oxidative damage, neurodegeneration, mitophagy, visual function

## Abstract

Mitochondrial autophagy plays a contributary role in the pathogenesis of retina degeneration (RD). ZYAN1 is a novel proline hydroxylase domain (PHD) inhibitor that can enhance the expression of hypoxia-inducible factor 1-alpha (HIF-1α). This study investigated whether ZYAN1 could alleviate progressive photoreceptor loss and oxidative damage in a pharmacologically induced RD model via the modulation of mitophagy. ZYAN1 was injected into the vitreous body of the RD model, and the retinal autophagy level was analyzed. The therapeutic effects of ZYAN1 were evaluated via a function examination, a morphological assay, in situ reactive oxygen species (ROS) detection, and an immunofluorescence assay. It was shown that the thickness of the outer nuclear layer (ONL) increased significantly, and visual function was efficiently preserved via ZYAN1 treatment. The mitochondria structure of photoreceptors was more complete in the ZYAN1-treated mice, and the number of autophagosomes also increased significantly. Membrane disc shedding and ROS overproduction were alleviated after ZYAN1 treatment, and the axonal cilia were more structurally intact. A Western blot analysis showed that the expression levels of the autophagy-related proteins LC3-B, Beclin-1, and ATG5 increased significantly after ZYAN1 treatment, while the expression of P62 was down-regulated. Moreover, the expression levels of HIF-1α and BNIP3 were up-regulated after ZYAN1 treatment. Therefore, an intravitreal injection of ZYAN1 can act as part of the pharmacologic strategy to modulate mitophagy and alleviate oxidative stress in RD. These findings enrich our knowledge of RD pathology and provide insights for the discovery of a therapeutic molecule.

## 1. Introduction

Retinal degeneration (RD) is a group of blinding eye diseases caused by heredity, phototoxicity, aging, and other environmental factors. These diseases are characterized by the progressive loss of photoreceptors and visual impairments [1]. The entire retinal ecosystem consists of rod and cone photoreceptors, neuroglia cells, and the retinal pigment epithelium (RPE), which are extremely sensitive to genetic or metabolic changes [2]. RD imposes a significant health burden, as more than 18 million individuals are affected [3]. Thus far, several etiological factors have been shown to be implicated in the pathophysiological mechanism of RD, including oxidative stress, inflammation, and aging-related impairments [4,5]. Due to the disease’s complex pathogenic background, the current existing therapeutic methods cannot completely arrest the photoreceptor death in RD. Autophagy plays a critical role in maintaining the homeostasis of photoreceptors. A reduced autophagy level can lead to the accumulation of damaged organelles; nonfunctional molecules; and toxic proteins, including lysosomal lipofuscin [6,7]. Autophagy acts as an important cellular clearance system in which cells utilize complex lysosome clearance processes to degrade and eliminate intracellular components [8,9]. During this process, damaged metabolites and organelles are removed from the cytoplasm. In the retina, RPE cells have the typical autophagy ability to continuously engulf the detached membrane disk of the photoreceptors [10]. Mitochondria are key organelles responsible for modulating energy metabolism and aerobic respiration. They significantly participate in the overproduction of reactive oxygen species (ROS) when their own redox balance is impaired [11]. However, mitochondria can be attacked by deleterious ROS, as their membrane proteins and DNAs are easily accessible [12]. Upon damage, mitochondria release pro-apoptotic proteins, such as cytochrome c (Cytc), apoptosis-inducing factor (AIF), and endonuclease G [13,14]. Therefore, it is well accepted that mitochondrial damage marks the so-called “point of no return”, meaning that cells with impaired mitochondria cannot survive for a long period of time [15].

Mitochondrial autophagy (mitophagy) acts as a selective degradation activity that can control the quality and quantity of mitochondria [16]. It has been shown that mitochondrial dysfunction, mitochondrial DNA (mt DNA) damage, and abnormal mitochondrial autophagy collectively contribute to RD pathology [17,18]. Therefore, modulating mitochondrial autophagy might be an effective strategy to maintain energy metabolism and inhibit ROS overproduction, thereby alleviating photoreceptor death [19,20,21,22].

Hypoxia-inducible factor (HIF) acts as a key transcription factor in the adaptive response to hypoxic environments [15]. HIF can reduce oxygen consumption in mitochondria by inhibiting the conversion of pyruvate to acetyl-CoA. It can also suppress mitochondrial biogenesis and activate mitochondrial autophagy [23]. Proline hydroxylase domain (PHD) is the most important protease that degrades HIF. HIF levels increase significantly when PHD activity is suppressed [24,25]. Therefore, PHD inhibitors may act as promising candidates for preventing metabolic stress [26]. Several lines of evidence lend support to the safety and therapeutic benefits of PHD inhibition in neurological disease [27,28,29,30]. ZYAN1 is a novel PHD inhibitor with strong permeability and tissue specificity. Pioneering reports have demonstrated that ZYAN1 analogs can enhance HIF-1α expression and consequently protect brain neurons from ischemic stroke [28,29]. However, whether ZYAN1 can modulate the HIF-1α expression in degenerating retinas remains an enigma.

Systemically administered sodium iodate (NaIO_3_) rapidly moves to the retina and selectively destroys RPE cells, leading to geographic atrophy, lipofuscin deposition, and progressive photoreceptor death [31,32,33]. Thus far, the NaIO_3_-induced RD model has been extensively used to study the pathology of and develop therapies for RD [34,35]. In this paper, we study the ZYAN1-induced effects on the photoreceptor survival and visual function in the RD model. We show that an intravitreal injection of ZYAN1 can enhance autophagy levels, reduce ROS production, and protect photoreceptors via the HIF-1α/BNIP3 signaling pathway. These findings may shed light on the discovery of a therapeutic molecule for RD.

## 2. Materials and Methods

### 2.1. Animals and Pharmacological Agents

**Figure 1:**
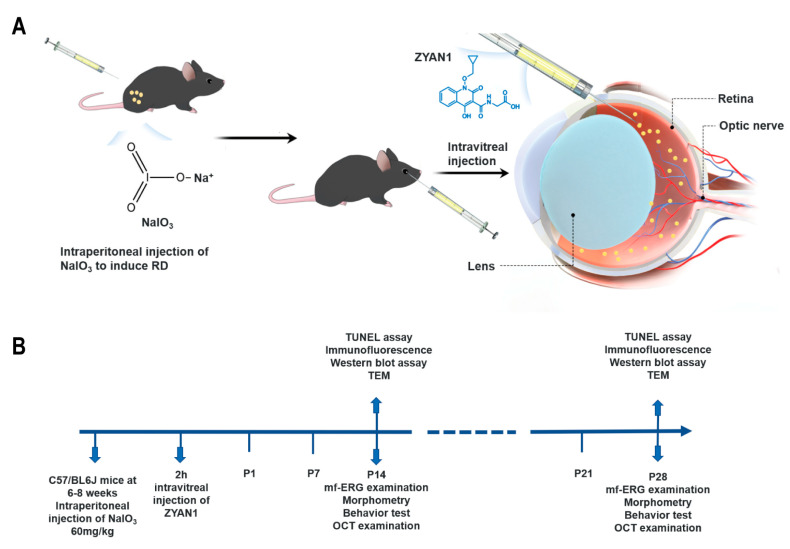
A schematic illustration of experimental protocols. (**A**) Intraperitoneal injection of NaIO_3_ to induce RD model, and injection of ZYAN1 was intravitreally delivered in the eyes of the RD model. (**B**) ZYAN1-administered mice were then subjected to a series of morphologic, functional, and mechanism analyses.

C57BL/6J mice (aged 6–8 weeks) were fed a normal diet at a temperature ranging from 21 to 25 °C, humidity ranging between 40 and 60%, and a light cycle of 24 h (8:00–20:00 light). The protocol of animal use was in accordance with the ARVO guidelines for ophthalmic and visual research. All animal experimental processes were approved by the Animal Ethics Review Committee of Zhengzhou University. NaIO_3_ (Sigma, 7681-55-2, Saint Louis, MO, USA) was stored at −20 °C and dissolved in phosphate-buffered saline (PBS) immediately before use. The mice were given a single intraperitoneal injection of NaIO_3_ solution at a dose of 60 mg/kg to induce RD. ZYAN1 (ChemeGen, C104783, Los Angeles, CA, USA) was firstly dissolved in DMSO and then injected into the vitreous using a 30 G microliter syringe (Hamilton Company, Model 701, Reno, NV, USA). All the experimental animals were randomly divided into 5 groups: (1) a normal control group; (2) an RD group: C57BL/6J mice were intraperitoneally injected with 60 mg/kg NaIO_3_ to establish the RD model; (3) an RD + ZYAN1 group: RD model received an intravitreal injection of 3 µL ZYAN1 (3.57 ng/µL); (4) an RD + vehicle group: RD model received an intravitreal injection of the same volume of DMSO; and (5) an RD + ZYAN1 + 3MA group: RD model received the ZYAN1 treatment and an intraperitoneal injection of 3-Methyladenine (3MA, at a dose of 30 mg/kg). The mice were sacrificed at different time points to harvest eye tissue for further analysis. Figure 1 is a schematic illustration of the experimental protocols.

### 2.2. Light/Dark Transition Behavior Test

Light/dark boxes (TopScan, CleverSys, Inc., Reston, VA, USA) were separated by a partition with a small hole underneath for the mouse to move through. A researcher adjusted the program settings of the computer’s topscan version 3.0 software. The monitoring device recorded the number of times that the mouse shuttled between the dark/light box and the time that they stayed in the dark box. After each test, the box was disinfected with the 75% alcohol.

### 2.3. Open-Field Behavior Test

The bottom surface of the open-field device (TopScan, CleverSys, Inc., Reston, VA, USA) was a square with the size of 40 cm × 40 cm. The surrounding environment was kept quiet to reduce possible influences on the mice. The mouse was placed in the middle of an open-field setup with a video detection system connected to a computer analysis system. The system synchronized and automatically recorded the autonomous activity of the mouse in the central and peripheral regions within a period of 10 min. GraphPad Prism Version 7.0 software (Graph Pad software, San Diego, CA, USA) was used to statistically analyze the total distance of mouse activity and the time spent in the central or periphery area.

### 2.4. Multifocal Electroretinography (mf-ERG) Examination

The mice were anesthetized via an intraperitoneal injection of 5% chloral hydrate (0.08 mL/10 g). Their eyes were dilated with compound tropicamide eye drops. Carbomer ophthalmic gel was used to prevent corneal dryness. Regional retinal function was measured using an image-guided mf-ERG system [36] (RETIscan, Roland Consult, Wiesbaden, Germany). RETIscan uses a confocal scanning laser ophthalmoscope (cSLO) to track the retinal region of interest when a stimulus is projected from a digital light processor (DLP). The mouse was positioned 1 to 2 mm in front of the cSLO device with a built-in light source for stimulus projection. A 3 mm gold ring was placed on the cornea to serve as a recording electrode. Meanwhile, a pair of subcutaneous silver needle electrodes served as reference and ground electrodes. The data obtained through the electrode were processed via the instrument’s supporting system to obtain digital signals and generate Excel tables. A total of 19 hexagonal stimuli were used to analyze the mean amplitude densities of the N-1 and P-1 waves corresponding to each ring [37].

### 2.5. Optical Coherence Tomography (OCT) and Fundus Photography

OCT (Heidelberg Engineering, Carlsbad, CA, USA) and fundus photography were used for in vivo retinal imaging. The mice were anaesthetized via an intraperitoneal injection of 5% chloral hydrate (0.08 mL/10 g). Tropicamide eye drops were used to dilate the eyes, and carbomer gel was used to prevent corneal dryness and cataracts. The mice were placed on a special small animal testing table in front of a corneal contact probe. The pupil of the mouse was aligned with the OCT probe, and the light angle was adjusted to obtain a clear retinal image. A circular scan was performed with the focus centered on the optic nerve papilla. The fundus photographs and OCT images were simultaneously captured on the exact retinal locus in a 30° circle surrounding the optic nerve head. After examination, the mouse was placed on a 37 °C thermal insulation blanket and resuscitated.

### 2.6. Hematoxylin and Eosin (H&E) Staining

Eyeballs were immediately removed and immersed in a fixation fluid. The cornea, lens, and iris tissues were removed under a stereo-microscope (Olympus, SZ61, Manila, Philippines). The retained optic cup was embedded in paraffin wax (SAKURA, TEC 5 EM JC-2, Tokyo, Japan) after conventional gradient alcohol dehydration, xylene clarity, and wax immersion (SAKURA, VIP-5-J R-JC2, Tokyo, Japan). Paraffin-embedded tissue was cut into 4 µm thick sections using a microtome (YAMATO, RX-860, Tokyo, Japan). Retinal sections were baked at 65 °C for 60 min, dewaxed with xylene, hydrated with gradient alcohol, soaked in pure water for 5 min, stained with hematoxylin for 20 s, and sealed with neutral resin. A fluorescence microscope (Olympus, BX53, Tokyo, Japan) was used to capture the retina images with a 4× or 20× objective lens. The outer nuclear layer (ONL) thickness in each image was measured with ImageJ Version 1.8.0 software (National Institutes of Health, Bethesda, MD, USA).

### 2.7. In Situ ROS Detection

Eyecups were harvested and embedded in an optimal cutting temperature compound (Tissue-Tek, Sakura, Torrance, CA, USA). Frozen sections were cut vertically into 6 µm thick sections using a Leica CM1900 cryostat (Leica, Wetzlar, Germany). ROS production was evaluated via dihydroethidium (DHE) (DHE, BBoxiProbe, BB-470516, China) staining. Briefly, a DHE activated oxygen fluorescence probe was diluted with pure water according to the number of samples, and a dye probe working solution was prepared. Each retinal section was incubated with DHE for 30 min at 37 °C and then rinsed three times with PBS for 5 min. Then, the retinal sections were photographed with a fluorescence microscope at 40× magnification and quantified via ImageJ Version 1.8.0 software.

### 2.8. Transmission Electron Microscope (TEM) Examination

Retinal tissue was peeled off under the stereo-microscope (Olympus, SZ61, Tokyo, Japan) and then quickly cut into small patches at a size of 1 mm^3^. The retinal patches were fixed in an electron microscope fixation solution (2.5% glutaraldehyde). Subsequently, the retinal tissue was washed with 0.1 M phosphate buffer PB (PH7.4) three time, and then fixed with 1% OsO4 at room temperature for 2 h. The retinal tissue was put in ethanol with a density gradient for upward dehydration. After being dehydrated with 100% acetone twice (15 min each time), the embedded tissue was polymerized in a 60 °C oven for 48 h, and a resin block was used for slicing. After being stained with citric acid, the sections were sent for TEM photography (Hitach, HT7800/HT7700, Tokyo, Japan).

### 2.9. Immunohistochemistry Assay

Experimental animals were sacrificed via cervical dislocation. Their eyeballs were enucleated, and the anterior segments were removed. The specimen was fixed in 4% paraformaldehyde for 2 h. Then, the specimen was dehydrated in a 30% sucrose solution overnight at 4 °C. Eyecups were embedded in optimal cutting temperature compounds (Tissue-Tek, Sakura, Torrance, CA, USA), and they were cut vertically into 6 µm thick sections using a Leica CM1900 cryothermostat (Leica, Wetzlar, Germany). Twenty slices were collected from each eyeball, and six were randomly selected for staining. Retinal sections were washed with PBS at room temperature. After immunohistochemical circles were drawn, sections were penetrated with 0.1% Triton X-100 for 20 min and then incubated with a blocking solution (3% BSA in PBS containing 0.1% Triton X-100) for 30 min at 37 °C. Then, they were incubated with primary antibodies, such as the autophagy markers rabbit anti-LC3B (Cell Signaling Technology, Danvers, MA, USA, 83506, 1:100), GFAP (Sigma, G3893, 1:200), and IBA1 (Abcam, Cambridge, UK, AB178847, 1:100), overnight at 4 °C. Retinal sections were rinsed three times with PBS and incubated with a secondary antibody (Alexa Fluor 488: Abcam, ab6785, ab150077) at room temperature for 1 h. The secondary antibodies were then washed with PBS, and the sections were sealed with an anti-fluorescence quencher containing DAPI (Invitrogen, P36971, Carlsbad, CA, USA). A clear visual field photo was taken on both sides of the optic nerve using a 40-fold mirror., The morphological indicators were analyzed via ImageJ software or GraphPad Prism version 7.0 software.

### 2.10. Terminal Deoxynucleotidyl Transferase Biotin-dUTP Nick End Labeling (TUNEL) Assay

Cell apoptosis was evaluated via an in situ cell death detection TUNEL kit (Roche, 11684817910, Mannheim, Germany). Frozen sections were washed three times with PBS for 5 min and then incubated with the TUNEL reaction mixture (TDT enzyme: fluorescent labeling solution = 1:9) for 1 h at 37 °C. Then, the retinal sections were incubated with 10 μL DAPI for 3 min at room temperature, sealed with anti-fluorescence quenching solution, and were photographed using the fluorescence microscope.

### 2.11. Western Blot Assay

Retinal tissue was placed in a solution containing RIPA lysate, a phosphatase inhibitor. After being centrifuged at 1800 r for 60 s, the specimens were left on ice for 10 min. The concentration of the extracted protein was determined using a BCA protein concentration assay kit (Beyotime, P0010, China). Then, the samples were adjusted to the same protein concentration (1 µg/µL) and denatured. Next, 10 µL of each sample containing an equal amount of protein was used for electrophoresis. The protein was transferred to a polyvinylidene difluoride (PVDF) membrane (Millipore, Billerica, MA, USA) in a transfer buffer. After blocking with 5% non-fat dry milk at room temperature for 2 h, the PVDF membranes were washed with Tris-buffered saline containing 0.1% Tween-20 (TBST) three times and incubated overnight at 4 °C with different primary antibodies, namely, HIF-1α (Cell signaling technology, 36169, 1:1000, USA), BNIP3 (Cell signaling technology, 3769, 1:1000, USA), LC3-B (Cell signaling technology, 83506, 1:1000, USA), ATG5 (Cell signaling technology, 12994T, 1:1000, USA), P62 (Abcam, ab109012, 1:10,000, UK), Beclin-1 (Cell signaling technology, 3738, 1:1000, USA), and GAPDH (glyceraldehyde 3-phosphate dehydrogenase) (Abcam, ab181602, 1:10,000, UK), overnight at 4 °C. After washing, the PVDF membrane was incubated with a goat anti-mouse IgG secondary antibody (Abcam, ab6789, 1:10,000, UK) or goat anti-rabbit IgG secondary antibody (Abcam, ab6721, 1:20,000, UK) at room temperature for 2 h. After three washes with TBST, ECL droplets were added to the PVDF membrane to evenly distribute its entire surface, and then it was transferred into a gel imaging system for photographing.

### 2.12. Statistical Analysis

All data are expressed as mean ± standard deviation (SD). They were statistically analyzed via a one-way ANOVA test using GraphPad Prism version 7.0 (Graph Pad software, San Diego, CA, USA). A *p*-value < 0.05 was considered statistically significant.

## 3. Results

### 3.1. Altered Autophagy Status in RD Model

**Figure 2:**
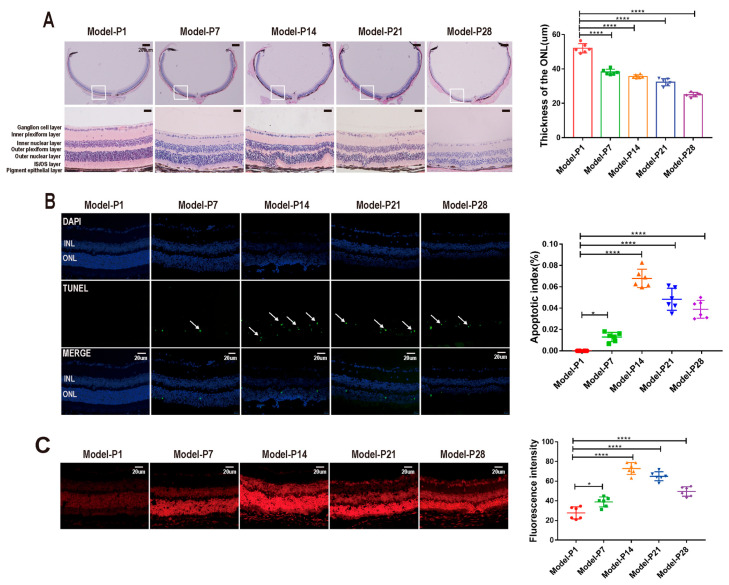
Morphological damage in the RD model. (**A**) The retinal morphology was damaged profoundly over time in the RD model. (**B**) The number of apoptotic cells in the RD model increased significantly. The apoptosis index peaked at P14. The white arrows represent TUNEL-positive cells. (**C**) The DHE staining showed that ROS accumulated in the retina of the RD model (one-way ANOVA multiple comparisons were analyzed; ns, * p < 0.05, **** p < 0.0001 for differences compared with P1; *n* = 6).

In the RD model, the retinal morphology was damaged profoundly over time (Figure 2A). Initially, at P1, the retinal architecture was intact with clear boundaries, and the retinal cells were densely arranged. Subsequently, the boundary of ONL became blurred, and the cell density prominently reduced. The thickness of ONL reduced significantly in a time-dependent manner (*p* < 0.0001, *n* = 6, Figure 2A). The TUNEL assay showed that the number of apoptotic cells in the retina of the RD model increased over time. The apoptosis index increased significantly and peaked at P14 (*p* < 0.001, *n* = 6, Figure 2B). Similarly, the fluorescence intensity of DHE staining peaked at P14 and then decreased until P28 (*p* < 0.001, *n* = 6), indicating that ROS accumulated in the retina of the RD model (Figure 2C).

**Figure 3:**
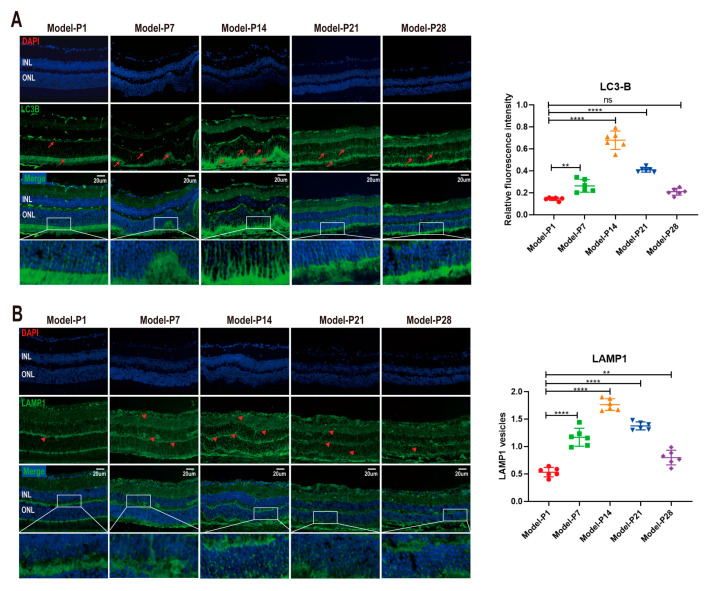
(**A**) LC3-B immunoreactivity in the retina sections of RD model. The LC3-B (green) staining was extremely faint at P1. However, LC3-B immunoreactivity increased at P7, as evidenced by the clusters of strongly stained small particles. Subsequently, at P14, the LC3-B immunostaining decreased over time. (**B**) The red arrows represent positive cells. The LAMP1-positive organelles in RD models were enlarged and looped at P7. The intensity of LAMP1-positive immunostaining peaked at P14 and then decreased progressively until P28. The red triangles represent positive cells (one-way ANOVA multiple comparisons were analyzed; ns, ** *p* < 0.01, **** *p* < 0.0001 for differences compared with P1; *n* = 6).

**Figure 4:**
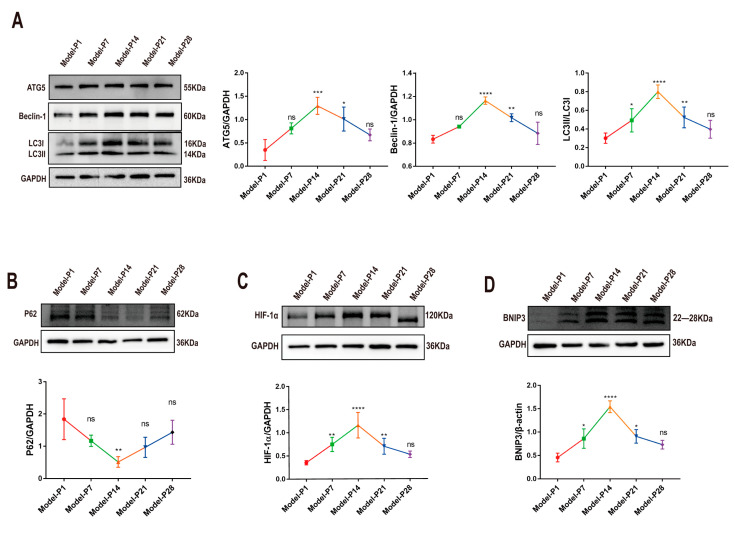
Expression levels of autophagy-related proteins in RD model. (**A**) Representative Western blot images of ATG5, Beclin-1, and LC3-B, and statistical line chart of gray value. (**B**) Representative Western blot images of P62 and statistical line chart of gray value. (**C**,**D**) Representative Western blot images of HIF-1α and BNIP3, and statistical line chart of gray value (one-way ANOVA multiple comparisons were analyzed; ns, * *p* < 0.05, ** *p* < 0.01, *** *p* < 0.001, **** *p* < 0.0001 for differences compared with P1; *n* = 6).

Microtubule-associated protein 1 light chain 3 (LC3) is the main biochemical marker of autophagy activation [38]. The LC3-B (green) staining was extremely faint in the retinal sections at P1. However, LC3-B immunoreactivity increased after P7, as evidenced by the clusters of strongly stained small particles. Subsequently, at P14, the LC3-B immunostaining decreased over time (Figure 3A). When autophagy was activated, cytoplasmic LC3 (LC3-I) hydrolyzed a small polypeptide and transformed it into an autophagy membrane type (LC3-II). The ratio of LC3-II/LC3I was used as a typical indicator to quantify the autophagy level. The Western blot assay showed that LC3II/LC3I expression increased significantly at P7 (*p* < 0.05, *n* = 6). Subsequently, at P14, LC3II/LC3I expression underwent a sharp reduction and then displayed a downward trend until P28 (Figure 4A). Lysosomal-associated membrane protein 1 (LAMP1) is a typical lysosomal marker. At P1, small spots of the LAMP1 immunostaining were distributed dispersedly in the retinal sections. Subsequently, the LAMP1-positive organelles in the RD models were enlarged and looped at P7, indicating that the cell debris degradation efficiency and the membrane recovery efficiency in lysozyme were extremely low. The intensity of the LAMP1-positive immunostaining peaked at P14 and then decreased progressively until P28 (Figure 3B).

The Bcl-2-interactin protein (Beclin-1) is a key regulator of autophagosome formation. The expression levels of Beclin-1 and autophagy-related 5 (ATG5) increased significantly between P7 and P14 (*p* < 0.001, *n* = 6, Figure 4A) and then reduced significantly until P28. Sequestosome1 (SQSTM1, also named P62) is a critical indicator of autophagy flux, which can be combined with a lysosome to form an autophagolysosome [39,40]. Its expression level is up-regulated when autophagy is inhibited. In the RD model, P62 expression peaked at P1 and decreased progressively until P14 (*p* < 0.0001, *n* = 6). Thereafter, P62 expression increased gradually until P28, indicating that dysfunctional autophagy may lead to lower autophagic flux and the accumulation of cytoplasmic debris (Figure 4B). As an important target of HIF-1α, BNIP3 can mediate autophagy activation. After modeling, the HIF-1α and BNIP3 expressions showed a transient increase and then reduced prominently at the later stages (Figure 4C,D).

### 3.2. Neuroglia Activation and Gliosis Reaction in the Retinas of RD Model

**Figure 5:**
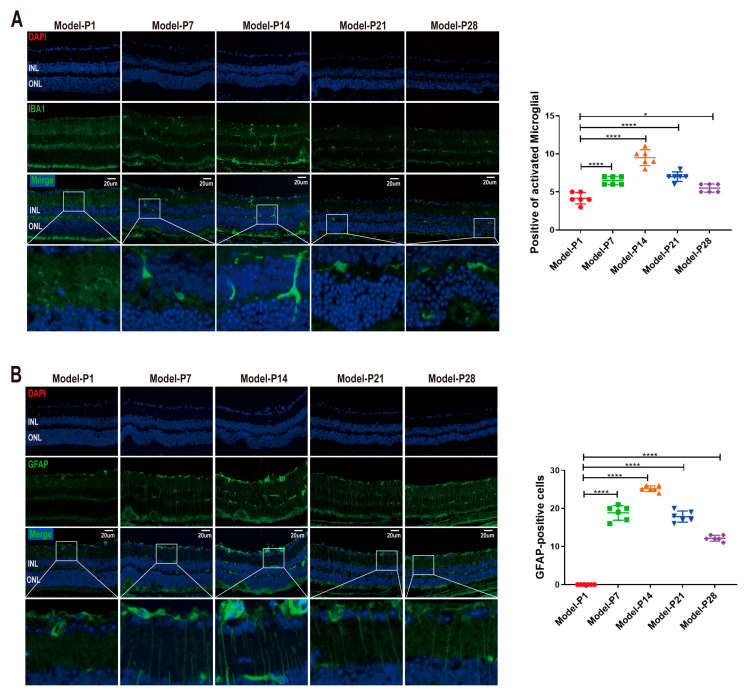
Activation of microglia and Müller cells in RD model. (**A**) The immunostaining intensity of IBA1 increased at the onset of RD. IBA1-positive cells were distributed in the inner nuclear layer with multi-branched shape. The activated microglia migrated into ONL at P14. (**B**) The GFAP expression was located exclusively at the feet of the ganglion cell layer (GCL) to form the inner limiting membrane (ILM) at P1. At P7, the distribution of GFAP immunostaining expanded to ONL. The number of GFAP-positive cells then decreased gradually until P28 (one-way ANOVA multiple comparisons were analyzed; * *p* < 0.05, **** *p* < 0.0001 for differences compared with P1; *n* = 6).

**Figure 6:**
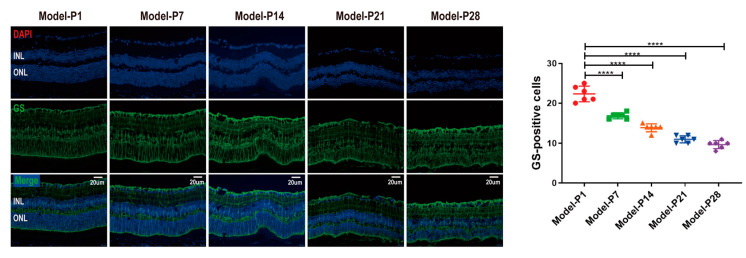
Activation of Müller cells in RD model. The positive signal of GS was uniformly expressed and neatly arranged at P1. The immunostaining intensity decreased gradually after P7, and its distribution was disordered and sparsely arranged. These results indicate that the Müller cells were activated in the retina of RD model (one-way ANOVA multiple comparisons were analyzed; **** *p* < 0.0001 for differences compared with P1; *n* = 6).

The immunostaining intensity of ionized calcium binding adapter molecule 1 (IBA1), a microglia-specific marker, increased at the onset of RD (*p* < 0.0001, *n* = 6). IBA1-positive cells were distributed in the inner nuclear layer (INL), with a multi-branched shape. However, the activated microglia migrated into ONL at P14 and showed an “amoeba” shape with hypertrophy and a reduced number of branches (Figure 5A). The glial fibrillary acidic protein (GFAP) is a specific marker of activated Müller cells. In RD models, the GFAP expression was distributed exclusively at the feet of the ganglion cell layer (GCL) to form the inner limiting membrane (ILM) at P1. Subsequently, at P7, the distribution of GFAP immunostaining expanded to ONL. The number of GFAP-positive cells increased significantly over time, peaked at P14, and then decreased gradually until P28 (*p* < 0.0001, *n* = 6, Figure 5B). Glutamine synthetase (GS) is a Müller cell-specific enzyme involved in neurotransmitter cycling and ammonia detoxification. Initially, at P1, the positive signal of GS was uniformly expressed and neatly arranged. However, GS immunostaining decreased gradually after P7, and its distribution was disordered and sparsely arranged (*p* < 0.0001, *n* = 6, Figure 6). These results indicate that the retinal neuroglia in the retina of the RD model were activated and resulted in glia hyperplasia.

### 3.3. ZYAN1 Alleviated Retinal Photoreceptor Damage in RD Model

**Figure 7:**
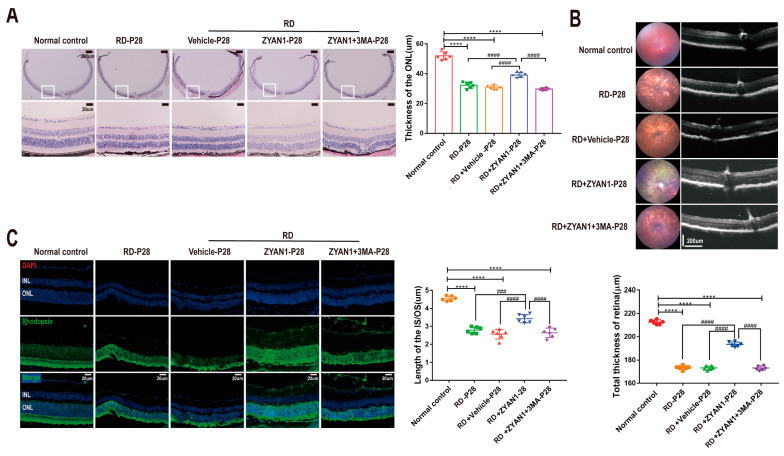
ZYAN1 alleviates the morphology damage in RD model at P28. (**A**) Retinal structure in the RD + ZYAN1 group was intact. The ONL in RD + ZYAN1 group was thicker than in RD group. (**B**) In OCT examination, the boundary between retinal layers was clear in the RD + ZYAN1 group, and the retinal thickness was significantly larger than in RD group. The scale of retinal lesions reduced prominently after ZYAN1 treatment. (**C**) Immunofluorescence staining of rhodopsin showed that the rod photoreceptors in the RD + ZYAN1 group were effectively preserved. The IS/OS length was significantly larger than in RD group (**** *p* < 0.0001 for differences compared with normal control group; ^###^ *p* < 0.001, ^####^ *p* < 0.0001 for differences compared with RD + ZYAN1 group, *n* = 6).

The retinal structure in the RD + ZYAN1 group was intact with a consolidated cell density. The ONL in the RD + ZYAN1 group was thicker than in the RD group (*p* < 0.0001, *n* = 6, Figure 7A and Figure S1A). The immunofluorescence staining of rhodopsin showed that the rod photoreceptors in the RD + ZYAN1 group were effectively preserved (Figure 7C and Figure S1C). As shown in the fundus photography, numerous yellow-light verruca-like lesions were detected in the RD model. The scale of the retinal lesions reduced prominently after ZYAN1 treatment (Figure 7B and Figure S1B). The OCT examination also showed the pigment disturbance, increased reflectivity, and decreased retinal thickness in the RD model. However, the boundary between the retinal layers was clear in the RD + ZYAN1 group, and the retinal thickness was significantly larger than in the RD group (*p* < 0.0001, *n* = 6, Figure 7B and Figure S1B). In particular, the ZYAN1-induced beneficial effects on retinal morphology were disrupted by 3MA, a specific autophagy inhibitor.

**Figure 8:**
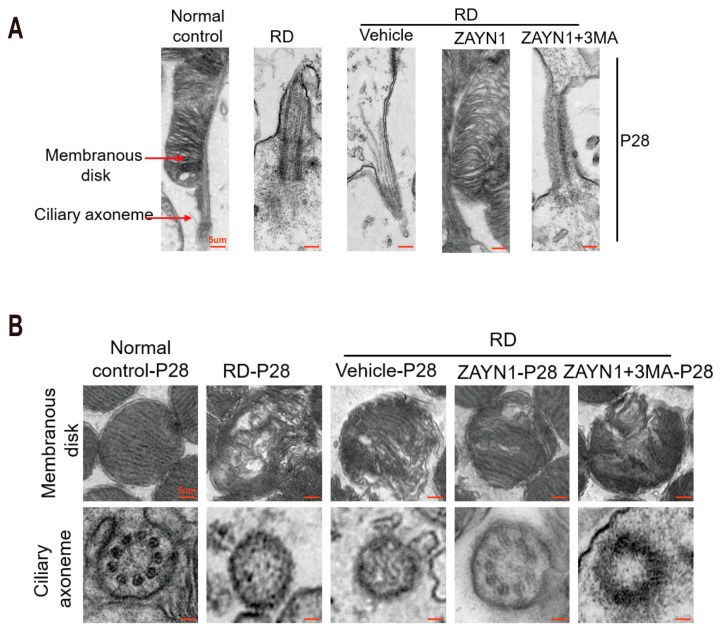
ZYAN1 mitigated the injury of ciliary axonemes and membranous disks in RD model mice at P28. (**A**) Transmission electron microscopy images of the longitudinal sections of photoreceptors. (**B**) Transmission electron microscopy images of the cross-sections of ciliary axonemes and membranous disks. After ZYAN1 treatment, the membrane disc shedding was ameliorated, and the structure of axonal cilia was relatively intact.

The photoreceptor cilium is essential for light sensation and phototransduction. The TEM observation showed that the membrane disc and axonal cilia in the RD model were abnormal. A large number of shaded membrane discs were found at the end of the outer segment. After ZYAN1 treatment, the membrane disc shedding was ameliorated, and the structure of the axonal cilia was relatively intact. The ZYAN1-induced protective effects on the microstructure of the photoreceptors were abolished by 3MA (Figure 8A,B and Figure S2A,B). These results suggest that ZYAN1 could enhance photoreceptor survival and alleviate the morphological damage in RD mice.

### 3.4. ZYAN1 Improved Visual Function and Behavioral Activity in RD Model

**Figure 9:**
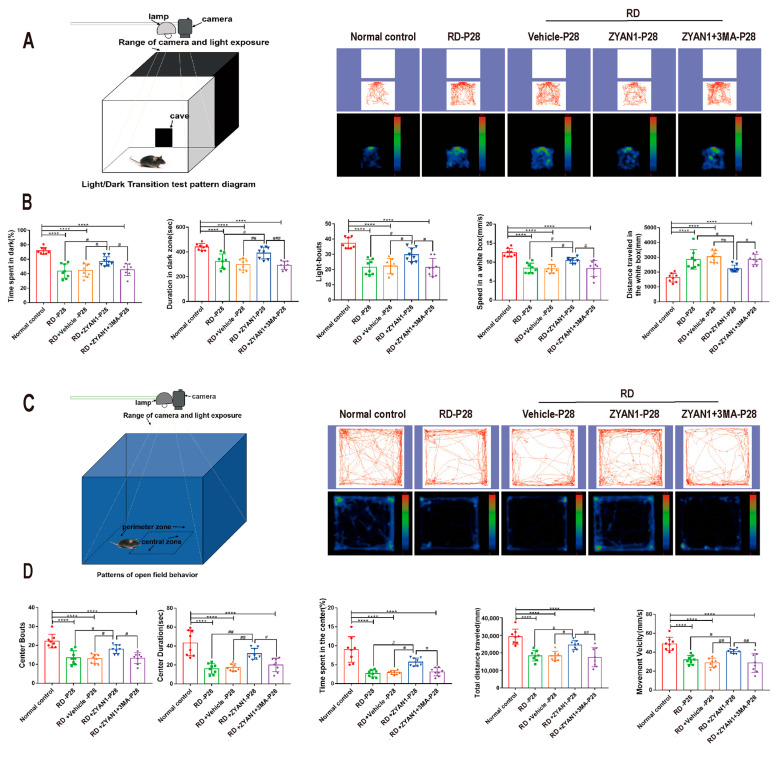
Effect of ZYAN1 on the behavioral activity of RD model. (**A**) Tracks and density maps of light/dark transition test. (**B**) The residence time in the dark box, the movement speed in the light box, and the shuttle times between two boxes in the RD + ZYAN1 group increased significantly compared with in RD group. (**C**) Track and density map of open-field test. (**D**) In the open-field experiment, the duration in the central area, the number of times entering the central area, the total moving speed, and the total distance in the open area increased significantly in the RD + ZYAN1 group compared with in RD group (**** *p* < 0.0001 for differences compared with normal control group; ^#^ *p* < 0.05, ^##^ *p* < 0.01, ^###^ *p* < 0.001 for differences compared with RD + ZYAN1 group, *n* = 8).

**Figure 10:**
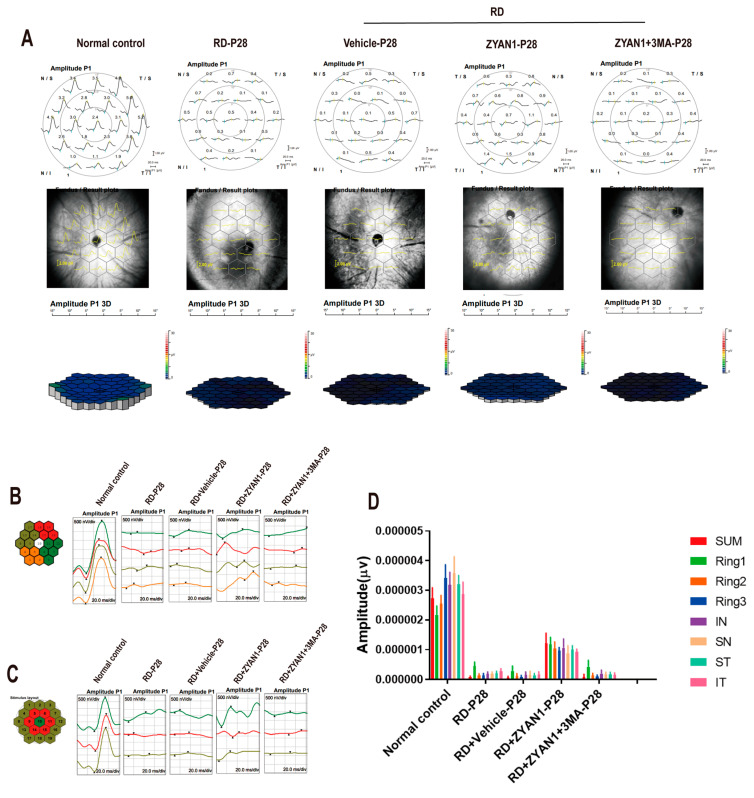
ZYAN1 can protect retinal function of RD mice at P28. (**A**) In mf-ERG examination, the amplitude of RD + ZYAN1 group increased significantly compared with that of RD group. (**B**) The mf-ERG amplitude increased comprehensively in all the ST, SN, IT, and IN quadrants after ZYAN1 treatment. (**C**) In the RD + ZYAN1 group, retinal amplitudes were significantly larger than in the RD group in ring1, ring2, and ring3. (**D**) mf-ERG amplitude histogram of different quadrants and rings.

The behavioral examination showed that ZYAN1 improved the visual function and behavior of the RD model. The behavioral parameters, including the residence time in the dark box, the movement speed in the light box, and the shuttle times between the two boxes, increased significantly in the RD + ZYAN1 group compared with the RD group (*p* < 0.05, *n* = 8, Figure 9A,B and Figure S3A,B). However, the movement distance in the light box reduced significantly in the RD + ZYAN1 group compared with in the RD group. In the open-field test, the duration in the central area, the number of times entering the central area, the total moving speed, and the total distance in the open area increased significantly in the RD + ZYAN1 group compared with in the RD group (*p* < 0.05, *n* = 8, Figure 9C,D and Figure S3C,D). In the mf-ERG examination, the amplitude of the RD + ZYAN1 group increased significantly compared with that of the RD group (*n* = 6, Figure 10A,D and Figure S4A,D). In particular, the mf-ERG amplitude increased comprehensively in all the ST, SN, IT, and IN quadrants after ZYAN1 treatment (Figure 10B and Figure S4B). In the RD + ZYAN1 group, the retinal amplitudes were significantly larger than those in the RD group in ring1, ring2, and ring3 (Figure 10C and Figure S4C). In particular, these improvements in behavioral activity and mf-ERG function could be blocked by 3MA.

### 3.5. ZYAN1 Restored Retinal Autophagy in RD Model

**Figure 11:**
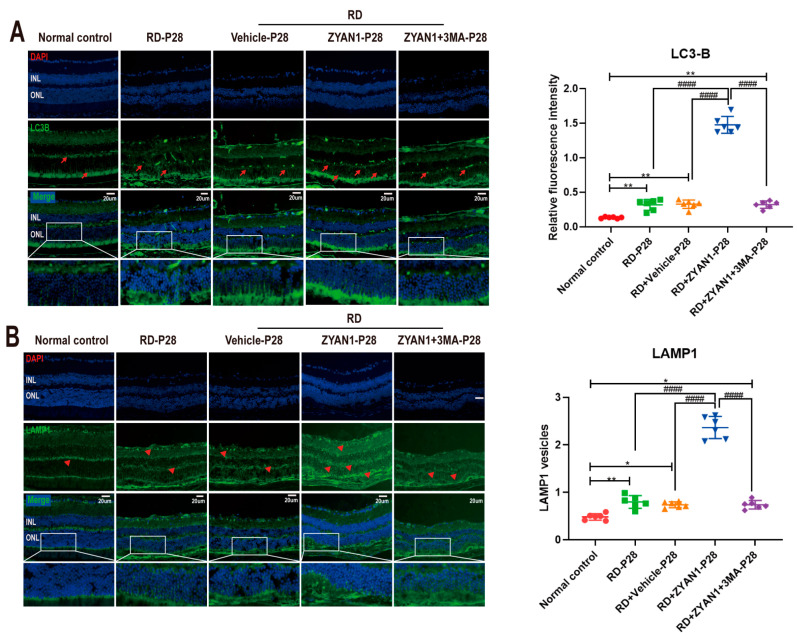
ZYAN1 enhanced the positive signal of LC3B and LAMP1 in RD models at P28. (**A**) Strong LC3-B spots were found in INL and ONL. Red arrows indicate positive cells. (**B**) Compared with RD model, more LAMP1-positive cells were detected in the retinas of RD + ZYAN1 group. Red triangle indicates positive cells (* *p* < 0.05, ** *p* < 0.01 for differences compared with normal control group; ^####^ *p* < 0.0001 for differences compared with RD + ZYAN1 group, *n* = 6).

**Figure 12:**
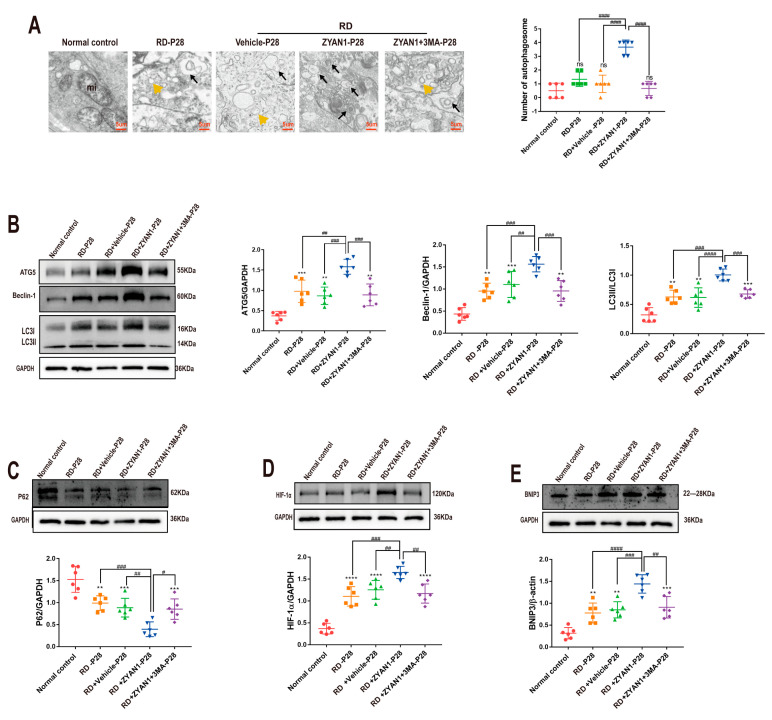
ZYAN1 enhances expressions of autophagy-related proteins and promotes the formation of autophagosome at P28. (**A**) TEM examination showed that the mitochondrial damage was ameliorated by ZYAN1 treatment, as evidenced by increased number of autophagosomes and reduced number of autophagy vacuoles. The black arrows represent autophagosomes; the yellow triangle represents autophagy vacuoles. (**B**) Western blot showed that the expression levels of ATG5, Beclin-1, and LC3-B in the retina of RD + ZYAN1 group increased at P28. (**C**) The expression level of P62 in RD + ZYAN1 group was significantly lower than that in RD model (**D**,**E**) Western blot showed that the expression levels of HIF-1α and BNIP3 protein in the retina of RD + ZYAN1 group increased compared with those in the RD group (ns, ** *p* < 0.01, *** *p* < 0.001, **** *p* < 0.0001 for differences compared with normal control group; ^#^ *p* < 0.05, ^##^ *p* < 0.01, ^###^ *p* < 0.001, ^####^ *p* < 0.0001 for differences compared with RD + ZYAN1 group, *n* = 6).

The immunofluorescence staining showed that the density of LC3-B immunoreactivity increased in the RD + ZYAN1 group. Strong LC3-B spots were found in INL and ONL, indicating that autophagy polymerization occurred in the retinas of the RD + ZYAN1 group (*n* = 6, Figure 11A and Figure S5A). Compared with the RD model, more LAMP1-positive cells were detected in the retinas of the RD + ZYAN1 group (*n* = 6, Figure 11B and Figure S5B). The Western blot assay showed that the expression levels of LC3-B, Beclin-1, and ATG5 were significantly higher in the RD + ZYAN1 group than in the RD model (Figure 12B and Figure S6B). However, the expression level of the P62 protein in the RD + ZYAN1 group was significantly lower than that in the RD model (Figure 12C and Figure S6C). In addition, the expressions levels of HIF-1α and BNIP3 increased significantly after ZYAN1 treatment (Figure 12D,E and Figure S6D,E). 3MA could disrupt the ZYAN1-induced effects on autophagy markers. The TEM examination showed that the number of autophagosomes in the retina of the RD group increased at P14 and then decreased sharply (Figure 12A and Figure S6A). In greater detail, the mitochondrial structure in the normal controls was intact, while the mitochondria in the RD group were swollen, with diffused crista and cytoplasmic vacuolation. However, the mitochondrial damage was ameliorated by ZYAN1 treatment, as evidenced by the increased number of autophagosomes (*p* < 0.01 and *p* < 0.05, respectively, *n* = 6) and the reduced number of autophagy vacuoles. These results suggest that ZYAN1 can enhance mitochondrial autophagy via the HIF-1α/BNIP3 pathway.

### 3.6. ZYAN1 Inhibited Neuroglia Activation and Alleviated Oxidative Stress

**Figure 13:**
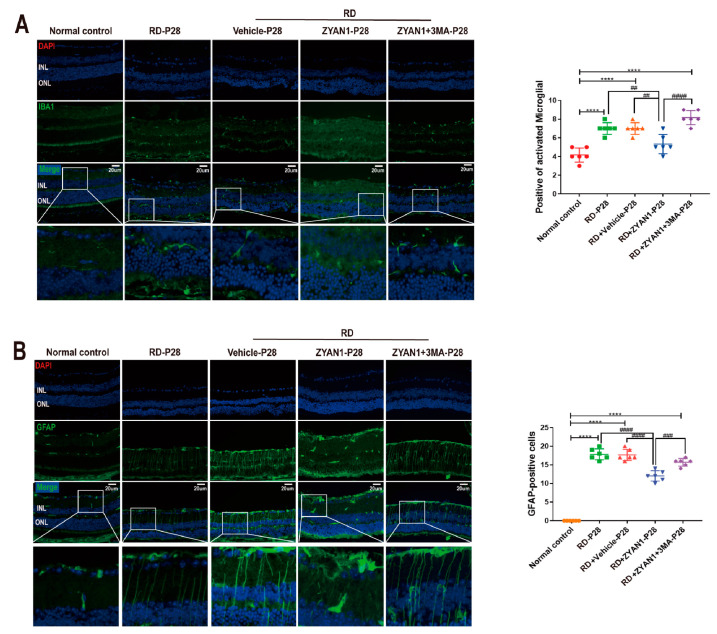
ZYAN1 inhibited the Müller and microglia activation in RD model at P28. (**A**) The number of IBA1-positive cells in RD+ZYA1N group reduced significantly compared with that in RD group. (**B**) The intensity of GFAP immunostaining in the RD + ZYAN1 group decreased compared with that in the RD group (**** *p* < 0.0001 for differences compared with normal control group; ^##^ *p* < 0.01, ^###^ *p* < 0.001, ^####^ *p* < 0.0001 for differences compared with RD + ZYAN1 group, *n* = 6).

**Figure 14:**
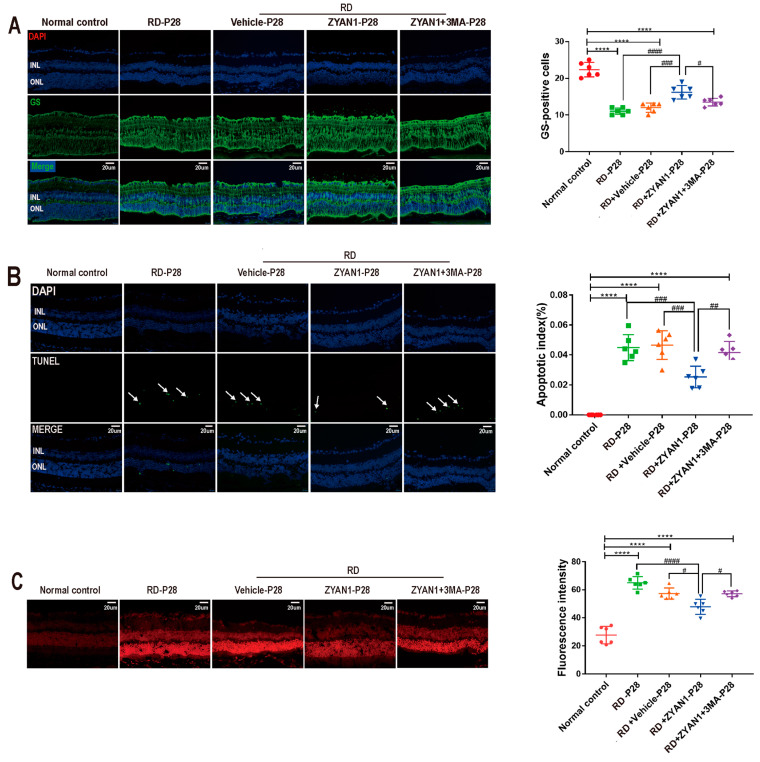
ZYAN1 mitigated photoreceptor cell death and ROS production in RD model at P28. (**A**) The density of GS immunostaining in RD + ZYAN1 group increased significantly compared with that in RD group. (**B**) The number of TUNEL-positive apoptotic cells in RD + ZYAN1 group reduced significantly compared with that in RD group. The white arrows represent TUNEL-positive cells. (**C**) The fluorescence intensity of DHE in RD + ZYAN1 group reduced significantly compared with that in RD group (**** *p* < 0.0001 for differences compared with normal control group; ^#^*p* < 0.05, ^##^ *p* < 0.01, ^###^ *p* < 0.001, ^####^ *p* < 0.0001 for differences compared with RD + ZYAN1 group, *n* = 6).

The immunofluorescence staining showed that the number of IBA1-positive cells in the RD + ZYA1N group reduced significantly compared with that in the RD group. The IBA1 immunostaining in the RD + ZYAN1 group was limited to the interior retina. The IBA1-positive cells in the RD + ZYAN1 group had multiple-branch protrusion, indicating that they were maintained at a static state (*p* < 0.01 and *p* < 0.001, respectively, *n* = 6, Figure 13A and Figure S7A). The intensity of the GFAP immunostaining in the RD + ZYAN1 group decreased compared with that in the RD group (*p* < 0.0001, *n* = 6, Figure 13B and Figure S7B). However, the intensity of the GS immunostaining in the RD + ZYAN1 group increased significantly compared with that in the RD group (*n* = 6, Figure 14A and Figure S8A). The number of TUNEL-positive apoptotic cells in the RD + ZYAN1 group was significantly smaller than in the RD group (*p* < 0.001, *n* = 6, Figure 14B and Figure S8B). Moreover, the fluorescence intensity of DHE in the RD + ZYAN1 group reduced significantly compared with that in the RD group (Figure 14C and Figure S8C). These results indicate that ZYAN1 could inhibit microglia activation and alleviate the oxidative stress in RD mice.

## 4. Discussion

Multiple genetic and environmental factors are involved in RD pathogenesis, including excessive oxidative stress, an impaired capacity of autophagy clearance, and chronic retinal inflammation [41,42,43]. Currently, there is no effective treatment for RD in clinical practice. There is an urgent need to deepen our understanding of RD pathophysiology and to develop integrated therapeutic strategies. At the initial stage of RD, the autophagy level increases to compensate for the oxidative stress and to scavenge the damaged organelles. During this time, the expression levels of autophagic factors, such as LC3, ATG9, and ATG7, increase significantly in retinal tissue [44]. However, at the advanced stages of RD, the expression levels of autophagic factors reduce markedly, leading to the accelerated progression of RD [45]. HIF-1 is a key heterodimer transcription factor in the oxygen homeostasis signaling system. It consists of an oxygen-regulated α subunit and a constitutionally expressed β subunit. The β subunit is expressed stably, and its expression is not affected by fluctuations in the oxygen concentration. Conversely, the expression of the α subunit is unstable, and it is regulated by PHD [46]. BNIP3 is a HIF-1-dependent gene that is responsible for promoting autophagic activity, thereby maintaining cellular homeostasis and facilitating the adaptation to stressful conditions [47]. In this study, we found that the autophagy level experiences a transitional increase and then declines sharply in the retina of the RD model. Autophagy can maintain cell survival by recycling abnormal proteins and removing abnormal cellular components through lysosomal-dependent degradation [48]. The decreased autophagy activity of the retinal cell leads to the accumulation of lipofuscins, the overproduction of ROS, protein aggregation, and inflammatory reactions [49]. In this context, ZYAN1, a novel prolyl hydroxylase inhibitor, is injected into the vitreous cavity to enhance mitochondrial autophagy. Dysautophagy is implicated in the occurrence and progression of RD [7,50,51]. Calpain activation can inhibit the autophagy of photoreceptors, thereby exacerbating the visual impairments in RD models. When calpain activation is blocked by SNJ-1945, autophagy levels are restored, and the photoreceptors survive [52]. Another in vitro study shows that cone cells with ATG5 gene defects are more susceptible to phototoxicity [53]. Rapamycin, an autophagy activator, alleviates the visible light-induced photoreceptor damage by alleviating endoplasmic reticulum stress [54]. Collectively, these findings suggest that autophagy should be modulated appropriately to maintain the survival of photoreceptors. Several pathways are involved in modulating the balance of mitochondrial autophagy. PINK1 can directly promote non-Parkin-dependent mitochondrial autophagy by collecting nuclear dot protein 52 and optineurin [55]. Another mitochondrial membrane protein, FUNDC1, can also enhance autophagy and mitigate photoreceptor damage [56,57]. In particular, the autophagy mediated by the HIF-1α/BNIP3 signaling pathway is implicated in the pathogenesis of RD. Studies have shown that the overexpression of BNIP3 can induce autophagy, while the knockout of BNIP3 aggravates anoxic injury in RPE cells [58]. Agreeing well with aforementioned reports, we showed that ZYAN1 can enhance the mitochondrial autophagy in retinal cells via the HIF-1α/BNIP3 pathway. ZYAN1 can act as an agonist with HIF-1α, which acts as an indispensable component of proteolytic mechanisms [59]. As the downstream target gene of HIF-1α, BNIP3 is specifically localized to mitochondria and participates in the autophagic clearance of damaged mitochondria [60]. HIF-1α silencing mitigates the expressions of BNIP3 and LC3-II in rat retina, resulting in exacerbated photoreceptor death [61]. However, the application of a PHD inhibitor in retinal detachment models significantly activates the HIF-1α/BNIP3 pathway, enhances mitochondrial autophagy, and thus alleviates histopathologic lesions [62]. As expected, the expression levels of the autophagy-related proteins LC3-B, ATG5, and Beclin-1 increase after ZYAN1 treatment, indicating that autophagy is activated in the retinas. Autophagy activation confers tremendous benefits on degenerating retinas, such as increased ONL thickness and tighter retinal structures with clear boundaries. The ZYAN1-treated mice also maintained a substantial proportion of visual function, as evidenced by the mf-ERG examination and behavioral test. These findings highlight the possibility that ZYAN1 may act as a therapeutic molecule to slow down the pathologic process of RD.

Oxidative stress causes a pigment disorder, the accumulation of intracellular lipofuscin, and the activation of apoptotic cascades [63]. As an important source of ROS, mitochondria are vulnerable to oxidative insults [64]. Abnormalities in the respiratory chain and mitochondrial membrane potential lead to the overproduction of ROS, which, in turn, attack mitochondria [65,66]. Mitochondrial autophagy is a form of selective autophagy highly sensitive to fluctuations in oxidative stress. Studies have shown that mitophagy can protect mitochondria from environmental insults [62,67]. In diabetic retinopathy, mitochondrial autophagy is down-regulated, and these damaged organelles cannot be cleared in a timely manner, resulting in the depletion of energy sources [68]. However, PINK1 can accumulate on the surface of the mitochondrial membrane to initiate mitochondrial autophagy and prevent the deterioration of retinopathy [69]. Accordingly, enhancing mitochondrial autophagy may act as a promising therapeutic strategy for RD. In this study, ZYAN1 treatment restored mitochondrial autophagy and inhibited ROS production. As shown in the TEM examination, ZYAN1 also restored the mitochondria structure and promoted the formation of autophagosomes. These anti-oxidative mechanisms may contribute to the ZYAN- mediated protection of degenerating retinas.

Microglia and Müller cells interact with each other to maintain the homeostasis of the retina. Under physiological conditions, microglia cells are mainly distributed in the inner plexiform layer (IPL) and outer plexiform layer (OPL), with a multiple-branched shape. When the retina is exposed to pathologic insults, microglia undergo morphological changes from branching to an “amoeba” shape, with an accompanying tendency to migrate to ONL [70]. These activated microglia impair the RPE’s ability to phagocytize the oxidized photoreceptor lipoproteins [71]. Moreover, activated microglia can produce a burst of cytotoxic molecules, including proteases, ROS, and nitric oxide, which can subsequently lead to an inflammatory microenvironment in the retina [72,73]. In particular, activated microglia can mitigate basal autophagy activity [74]. Therapeutically, ZYAN1 suppresses the microglia activation and the downstream inflammatory response in RD mice. This may be an important mechanism underlying the restored autophagy activity in the ZYAN1-treated mice. Additionally, ZYAN1 can ameliorate the retinal gliosis in RD mice. A retinal gliosis scar is formed by Müller cell hypertrophy and proliferation [75]. During the gliosis process, GFAP immunostaining is up-regulated, especially in a radial direction from the foot of Müller cells [71]. Müller cells further communicate with the activated microglia and promote the formation of drusen [76]. In this context, the reactive gliosis may impose a thorny challenge for any therapy. Encouragingly, ZYAN1 can inhibit the Müller cell activation and ameliorate the gliosis response in the RD model. This should be considered an advantage of ZYAN1 treatment.

Although we demonstrated that ZYAN1 could activate mitochondrial autophagy via the HIF-1α-BNIP3 pathway, the causal relationship among mitophagy, HIF-1α/BNIP3 signaling, and degenerating retinas has not been well established. In this context, further studies are necessary to elucidate the underlying mechanism with the assistance of some protein antagonists.

## 5. Conclusions

ZYAN1 can protect the photoreceptors in the RD model via the HIF-1α/BNIP3 pathway. Mitochondrial autophagy in retinal cells is recovered by ZYAN1, while the oxidative stress and glia hyperplasia are inhibited. These beneficial effects can be translated into prominent improvements in visual function and behavioral activity. Further refinements of these findings would enrich our knowledge of RD and contribute to the development of pharmacological therapy.

## Data Availability

All of the data are contained within the article.

## Supplementary Materials

The following supporting information can be downloaded at: https://www.mdpi.com/article/10.3390/antiox12111914/s1, Figure S1: ZYAN1 alleviates the morphology damage in RD model at P14; Figure S2: ZYAN1 mitigated the injury of ciliary axonemes and membranous disks in RD model mice at P14; Figure S3: Effect of ZYAN1 on the behavior of RD model in light/dark transition test and open-field transition at P14; Figure S4: ZYAN1 can mitigate the impairments of retinal function in RD mice at P14; Figure S5: ZYAN1 enhanced the positive signal of LC3B and LAMP1 in RD models at P14; Figure S6: ZYAN1 enhanced retinal autophagy in RD model at P14; Figure S7: ZYAN1 inhibited the Müller and microglia activation in RD model at P14; Figure S8: ZYAN1 reduces reactive gliosis, mitigated photoreceptor cell death, and ROS production in RD model at P14.
