# Supplementary material for: Intravitreal Injection of ZYAN1 Restored Autophagy and Alleviated Oxidative Stress in Degenerating Retina via the HIF-1α/BNIP3 Pathway
Source: Antioxidants (Basel). 2023 Oct 26;12(11):1914. doi: 10.3390/antiox12111914 (PMC10669006; doi:10.3390/antiox12111914)
Supplement: Supplementary file 1 [file antioxidants-12-01914-s001.zip › antioxidants-2624714-supplementary.pdf]

antioxidants

MDPI

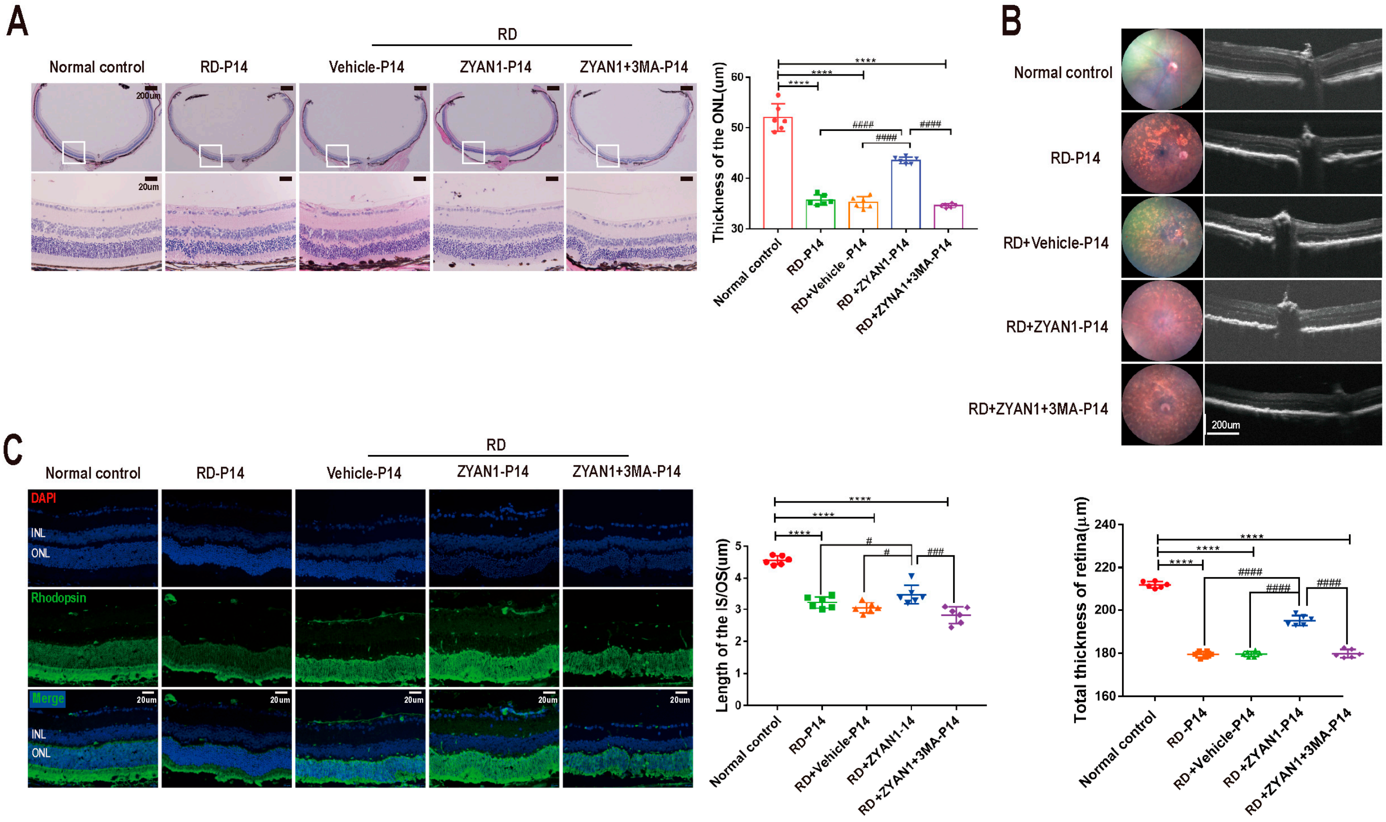

Figure S1. ZYAN1 alleviates the morphology damage in RD model at P14.

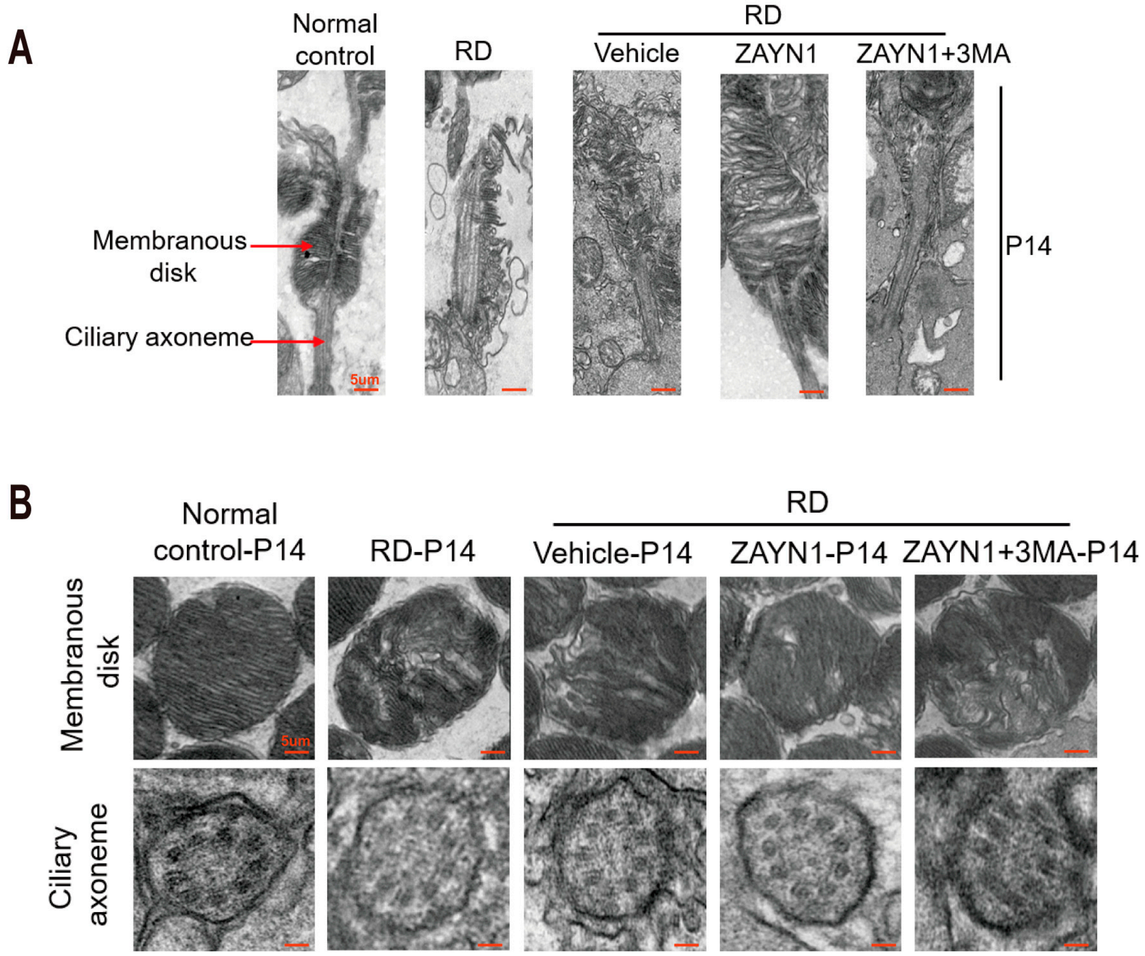

Figure S2. ZYAN1 mitigated the injury of ciliary axonemes and membranous disks in RD model mice at P14.

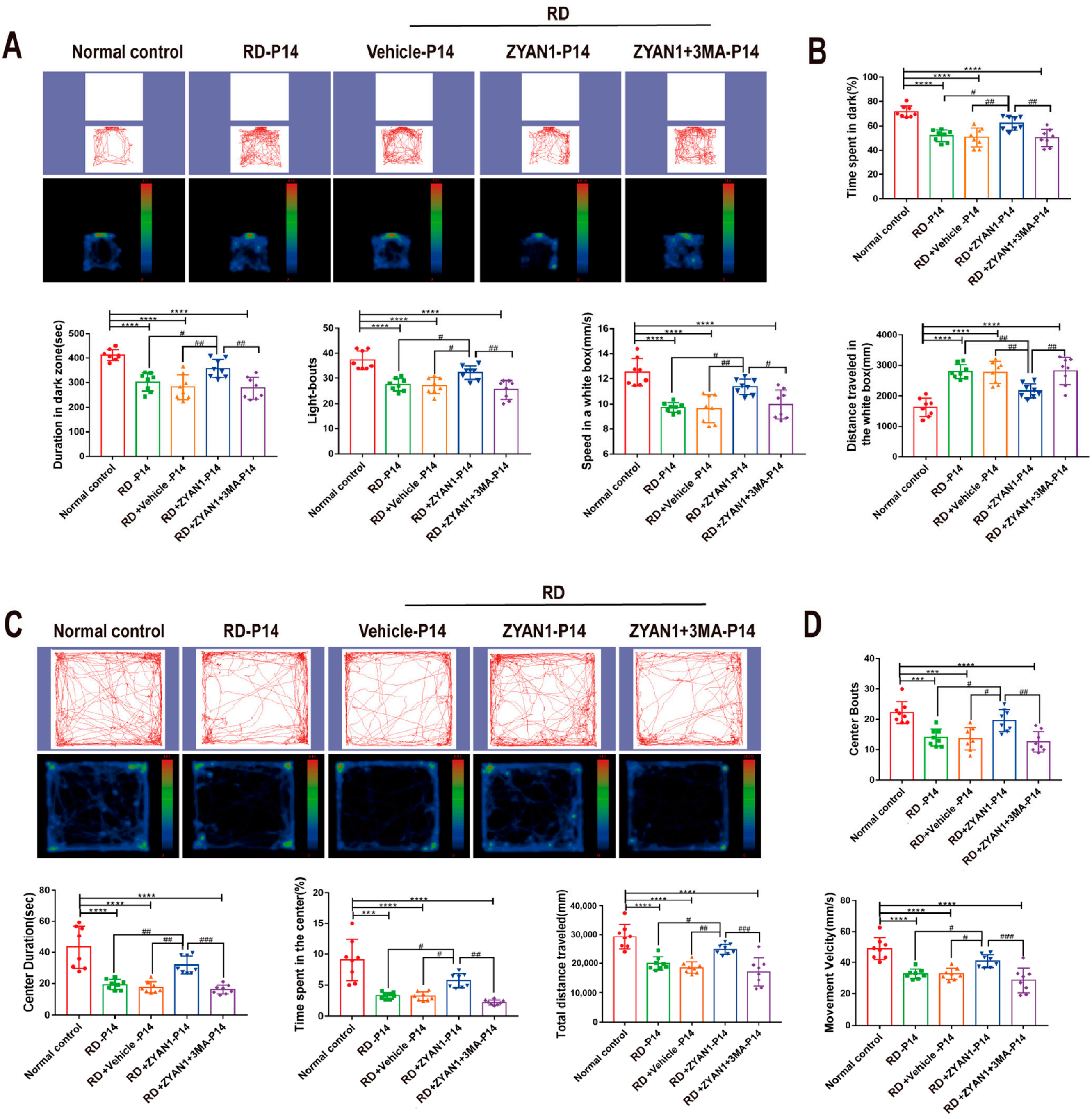

Figure S3. Effect of ZYAN1 on the behavior of RD model in light/dark transition test and open-field transition at P14.

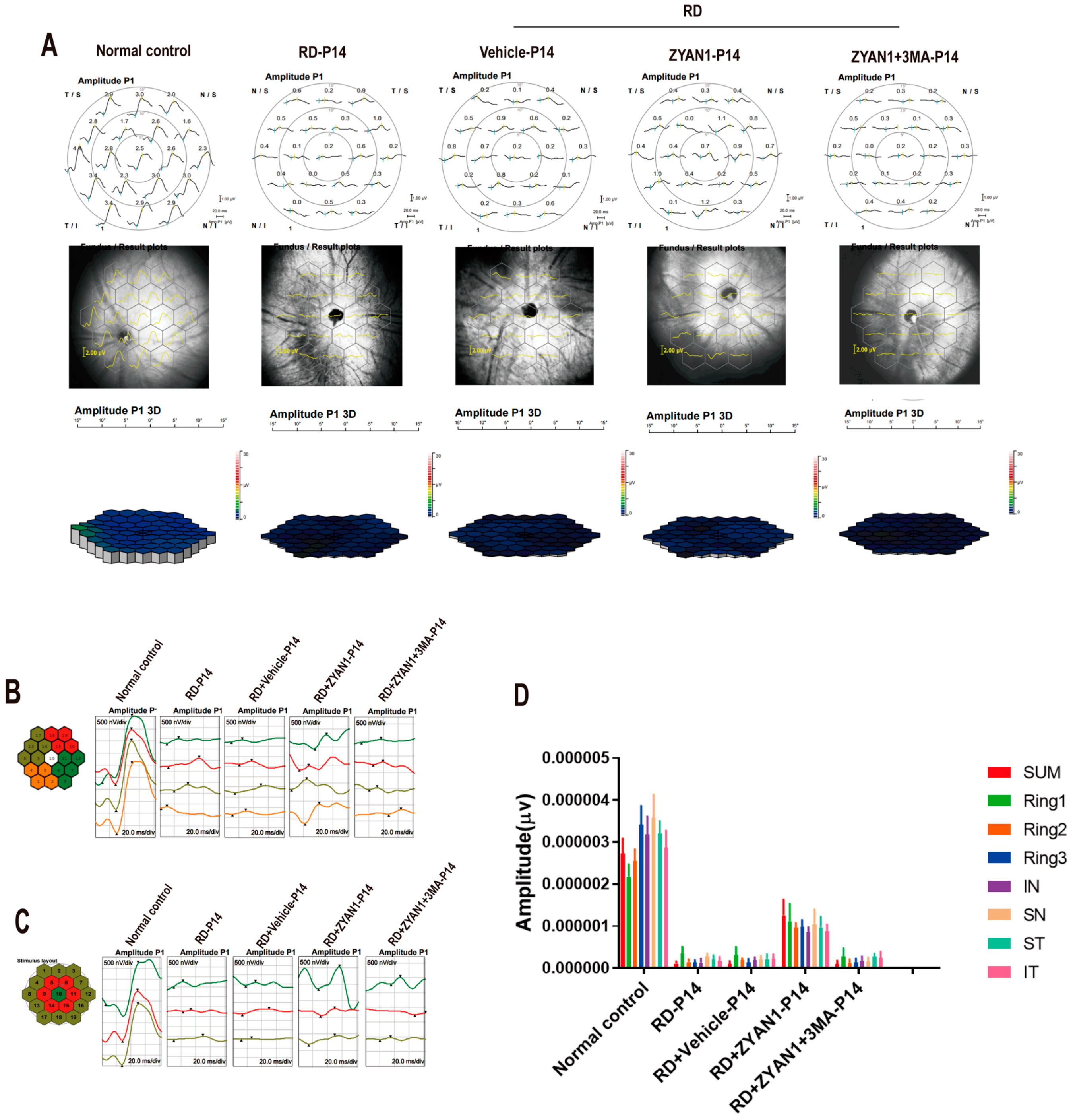

Figure S4. ZYAN1 can mitigate the impairments of retinal function in RD mice at P14.

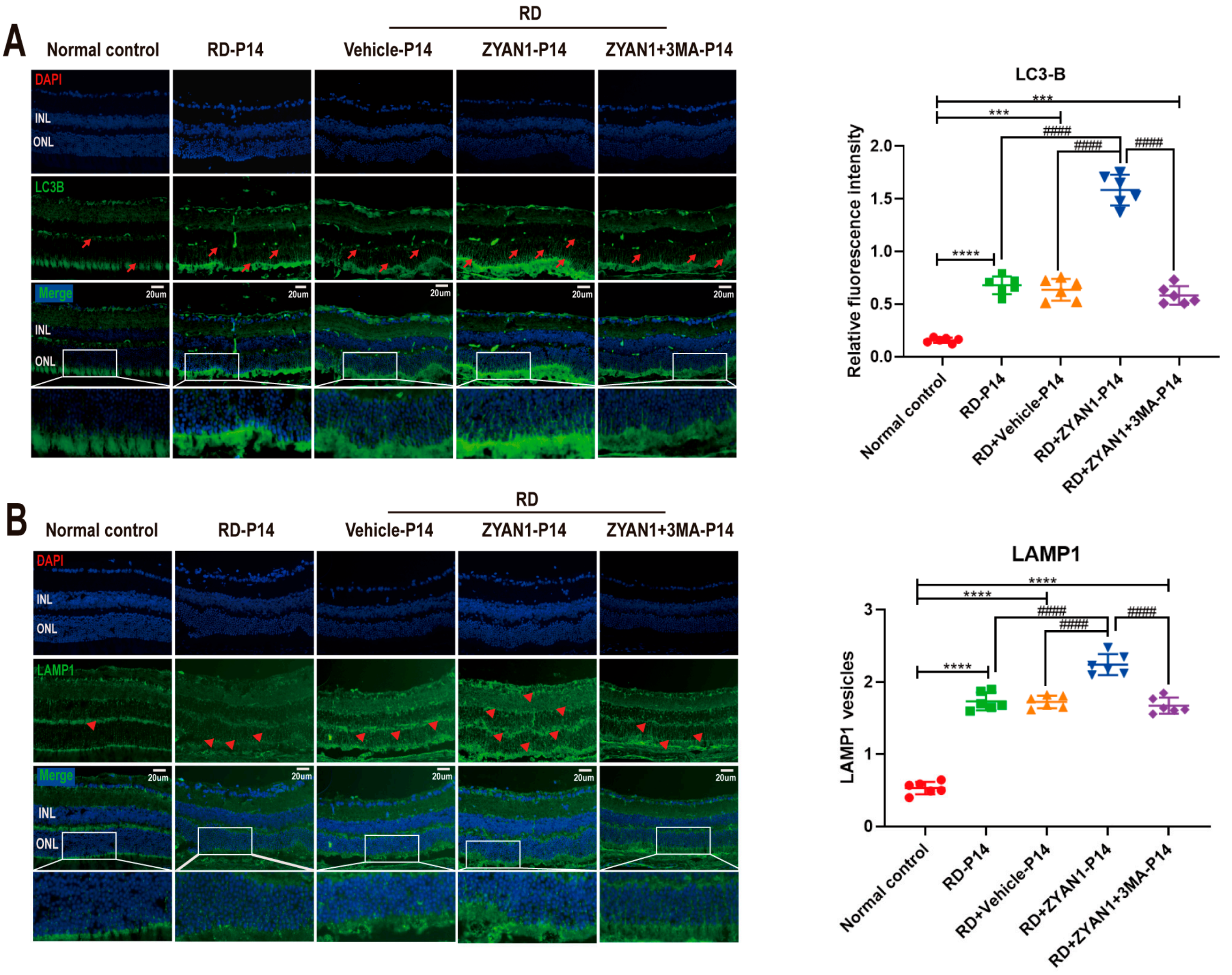

Figure S5. ZYAN1 enhanced the positive signal of LC3B and LAMP1 in RD models at P14.

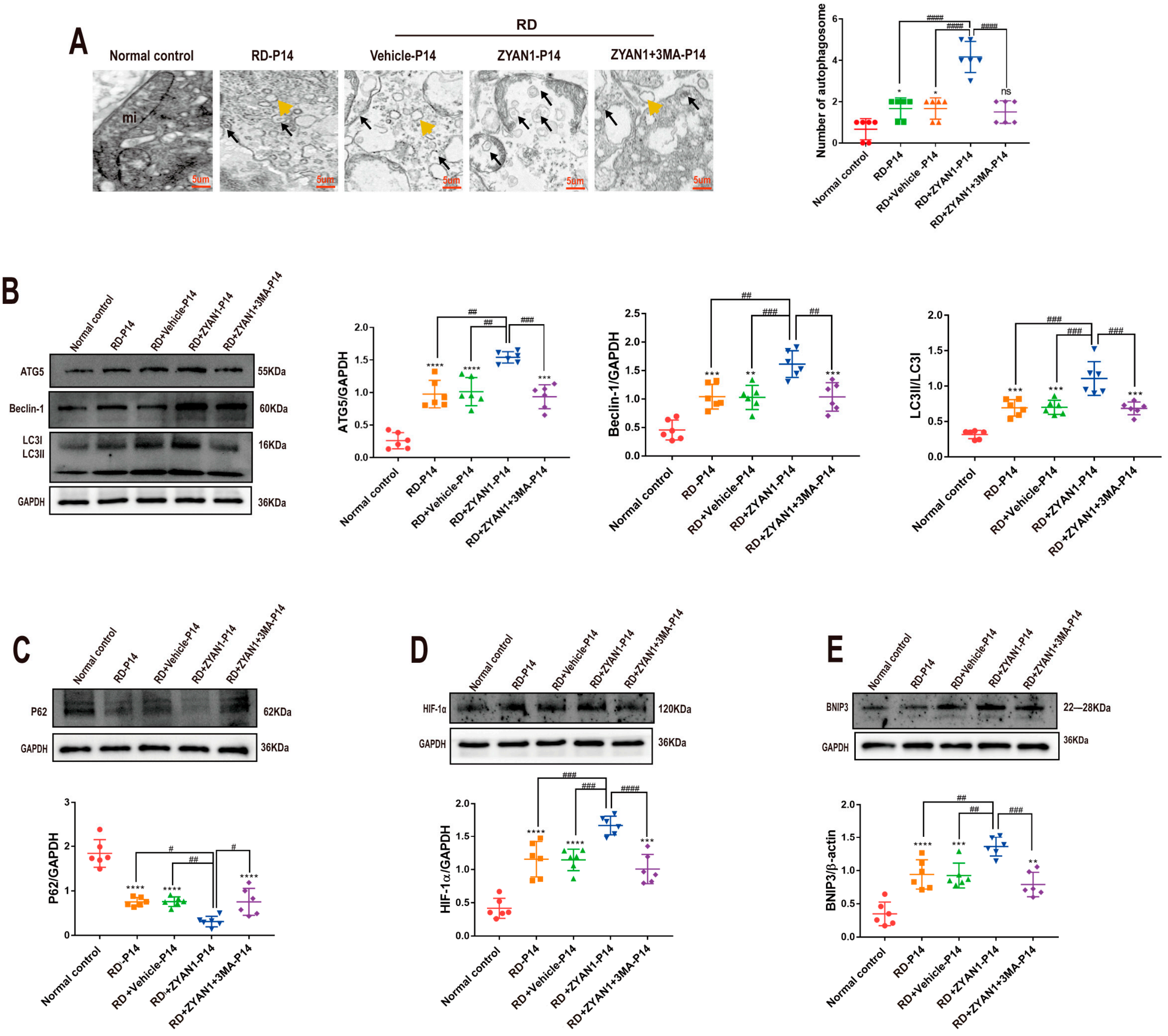

Figure S6. ZYAN1 enhanced retinal autophagy in RD model at P14.

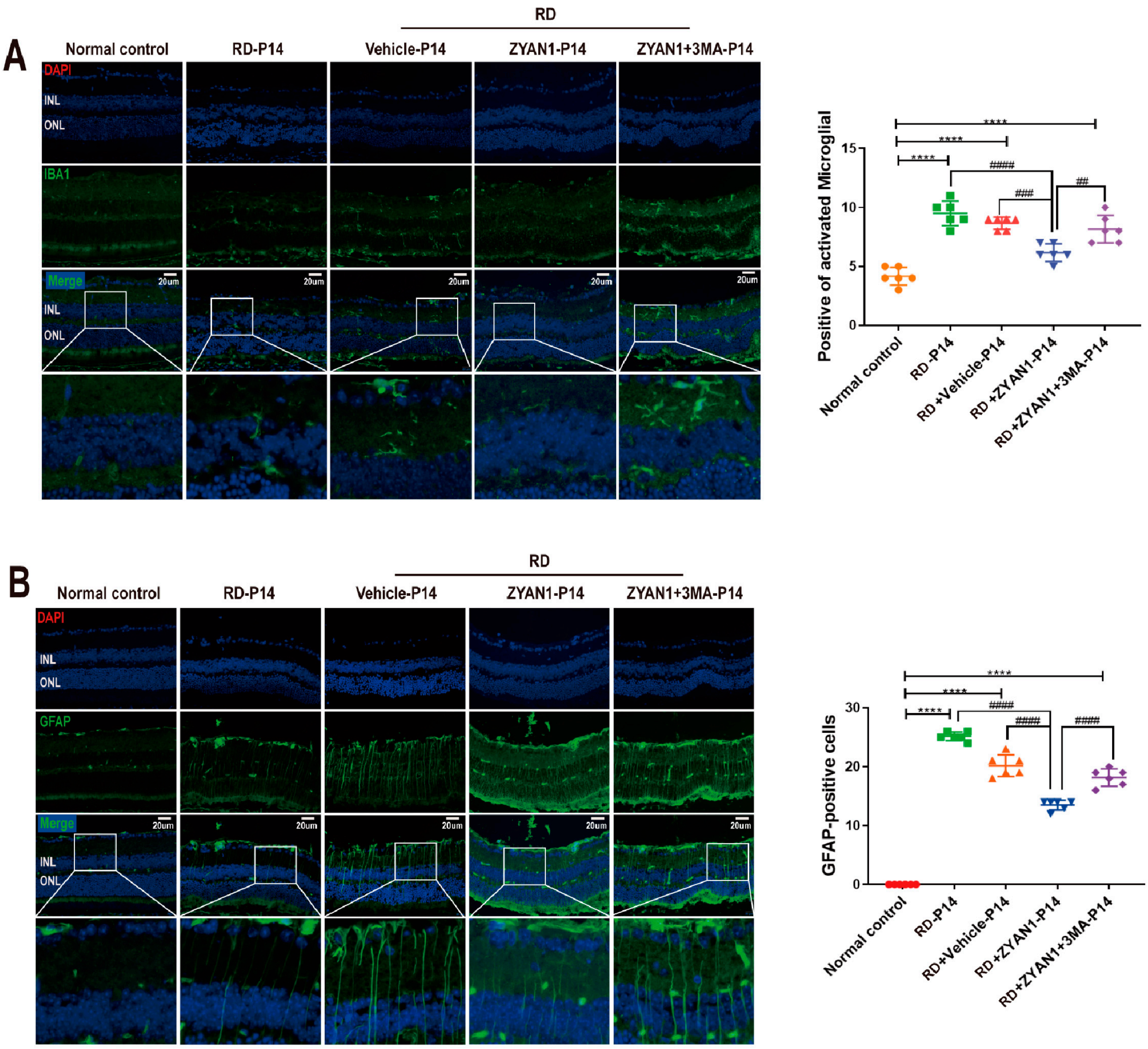

Figure S7. ZYAN1 inhibited the Müller and microglia activation in RD model at P14.

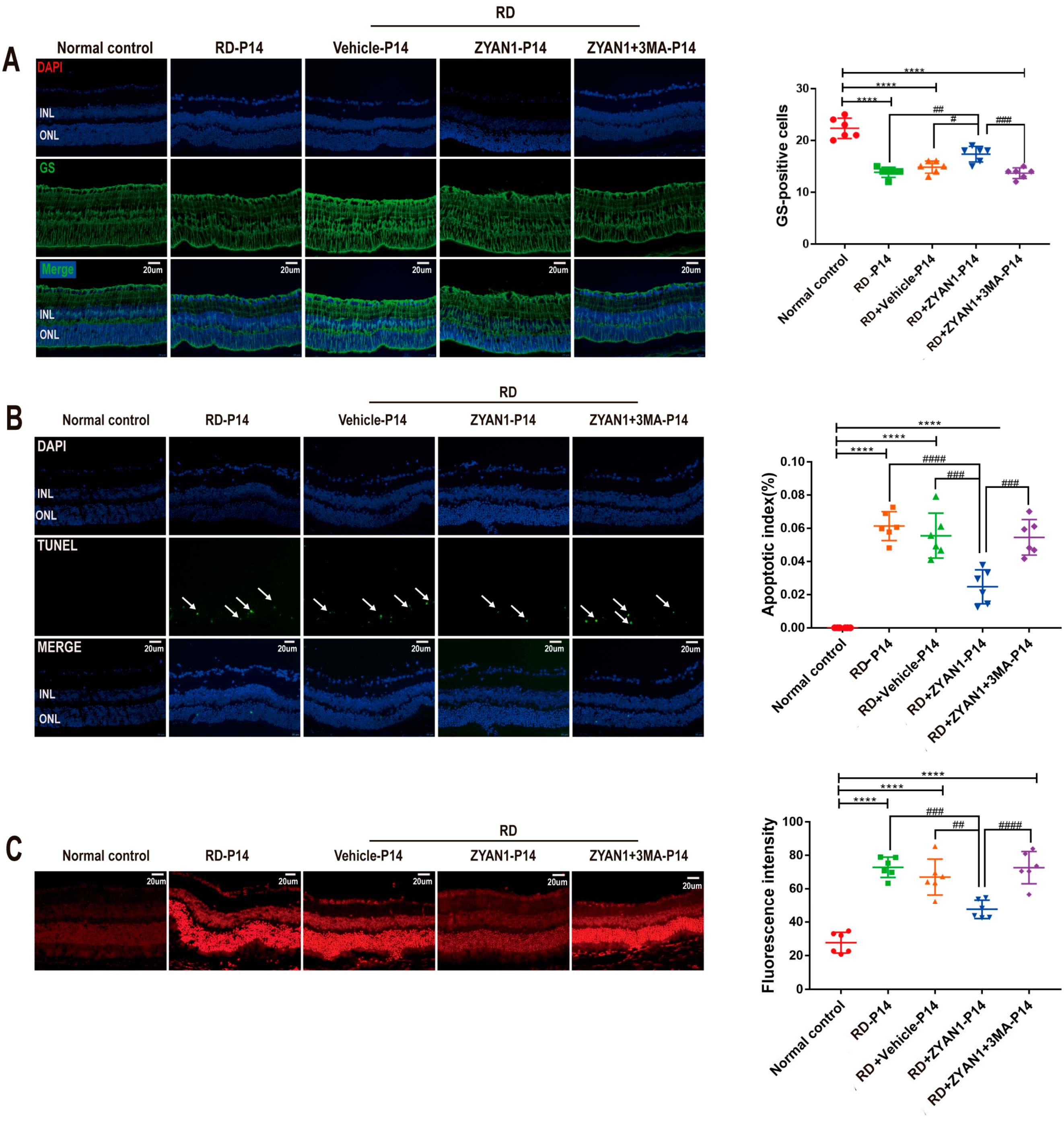

Figure S8. ZYAN1 reduces reactive gliosis, mitigated photoreceptor cell death, and ROS production in RD model at P14.
